# Imported Melioidosis, Israel, 2008

**DOI:** 10.3201/eid1511.090038

**Published:** 2009-11

**Authors:** Avivit Cahn, Benjamin Koslowsky, Ran Nir-Paz, Violeta Temper, Nurit Hiller, Alla Karlinsky, Itzhak Gur, Carlos Hidalgo-Grass, Samuel N. Heyman, Allon E. Moses, Colin Block

**Affiliations:** Hadassah–Hebrew University Hospital, Mount Scopus, Jerusalem, Israel (A. Cahn, B. Koslowsky, N. Hiller, S.N. Heyman); Hadassah–Hebrew University Medical Center, Ein Kerem, Jerusalem (R. Nir-Paz, V. Temper, C. Hidalgo-Grass, A.E. Moses, C. Block); Hebrew University School of Public Health, Jerusalem (A. Karlinsky, I. Gur); Clalit Health Services, Tel Aviv, Israel (A. Karlinsky, I. Gur)

**Keywords:** Melioidosis, Burkholderia pseudomallei, bacteria, biohazards, laboratory infection, chemoprophylaxis, Israel, Thailand, migrant workers, dispatch

## Abstract

In 2008, melioidosis was diagnosed in an agricultural worker from Thailand in the southern Jordan Valley in Israel. He had newly diagnosed diabetes mellitus, fever, multiple abscesses, and osteomyelitis. *Burkholderia pseudomallei* was isolated from urine and blood. Four of 10 laboratory staff members exposed to the organism received chemoprophylaxis, 3 of whom had adverse events.

Melioidosis, which is caused by *Burkholderia pseudomallei*, is endemic to some areas of Southeast Asia and northern Australia (*1**,**2*). Recent data indicate that it is now endemic to most of the Indian subcontinent, southern People’s Republic of China, Hong Kong, Taiwan, Papua New Guinea, and other regions (*3*). Most cases reported in other regions were acquired during residence in or travel to disease-endemic regions.

Thailand is a popular destination for backpackers from Israel. Importation of melioidosis has long been anticipated as a potential problem because many persons from Thailand are employed in Israel. Although most infections are asymptomatic (*2*) and usually occur in persons <6 years of age in disease-endemic areas, clinical, often life-threatening, disease most frequently affects adults who have underlying predisposing conditions, especially type 2 diabetes. Incubation period differs according to manner of exposure and size of inoculum and may be short (1 day to 2–3 weeks). However, because the organism has a proclivity for latency (*4*), the disease may appear after months or many years (*2**,**4*). Melioidosis is often manifested as pneumonia, but its hallmark is disseminated abscess formation in viscera, skin, soft tissue, and bone. We report a case of imported melioidosis (*5*) and management and consequences of chemoprophylaxis among laboratory staff exposed to *B*. *pseudomallei*.

## The Case

A 32-year-old man from Thailand was referred to the emergency department of Hadassah–Hebrew University Hospital at Mount Scopus on July 31, 2008, with newly diagnosed diabetes and fever. He reported 2–3 weeks of fatigue, chills, night sweats, and a weight loss of ≈25 kg in the past 2 months. Two large subcutaneous abscesses had developed over the past several months. The first abscess, in the right axilla, had been drained in May 2008. The second abscess, in the upper right abdominal wall, had been drained in July 2008. Pus was not submitted for culture.

The patient, an agricultural worker, arrived in Israel in November 2007 and was employed at a rural settlement in the southern Jordan Valley. He came from a village in northeastern Thailand where he had worked in rice and sugar cane farming. At the time of admission, he appeared ill and was febrile (39.0°C). Physical examination showed mild cervical lymphadenopathy, nontender hepatomegaly, and healing wounds from the abscess drainage procedures. Laboratory results showed hyperglycemia (glucose level 19.4 mmol/L), normocytic anemia with normal leukocyte and platelet counts, an erythrocyte sedimentation rate of 105 mm/h, and moderately elevated levels of alkaline phosphatase and γ-glutamyl transferase. Kidney function was normal. Urinalysis showed leukocyturia and nitrites. Multiple abscesses were seen in the spleen, lungs, superior pole of the right kidney, prostate gland, and right foot (Figure). A bone scan confirmed osteomyelitis of the right medial malleolus, calcaneus, and first metatarsus.

**Figure:**
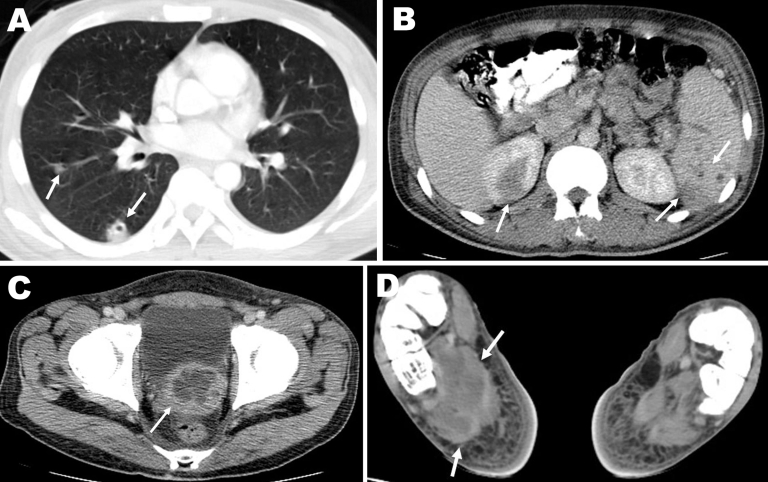
Computed tomography images showing multiple abscesses in the right lung (A), spleen and upper pole of the right kidney (B), prostate gland (C), and plantar aspect of the right foot (D) (arrows) of the patient.

A diagnosis of melioidosis had been considered from the outset in view of clinical findings of multiple abscesses in a patient with diabetes from a disease-endemic area. A blood culture (BacTec+ Aerobic/F; Becton, Dickinson and Company, Sparks, MD, USA) yielded a nonfermentative, oxidase-positive, motile, colistin-resistant, gram-negative bacilli that showed dry wrinkled colonies. The API 20 NE profile (API; bioMérieux, Durham, NC, USA) was 1556577, which suggested esculin-positive *B*. *pseudomallei*.

Molecular confirmation was achieved by bidirectional sequencing of a 1.7-kb amplicon specific for the 16S rRNA gene, which was amplified by PCR and primers F229 and R1908 (*6*). Sequencing (National Center for Biotechnology Information accession no. FJ426359) with the same primers showed the known single basepair transition (C/T) at position 75 that distinguishes *B*. *pseudomallei* from *B*. *mallei*, the agent of glanders (*6*).

Susceptibility to trimethoprim/sulfamethoxazole was confirmed. MICs were 0.75 mg/L for trimethoprim and 14.25 mg/L for sulfamethoxazole (Etest; AB Biodisk, Solna, Sweden). A urine culture was positive for *B*. *pseudomallei*, and throat and splenic abscess aspirate cultures were negative.

The patient’s first abscess developed ≈6 months after his arrival in Israel, which attests to a long incubation period or prolonged latency after initial asymptomatic infection. New-onset diabetes might have unmasked a preexisting latent infection. He had no history of an illness compatible with melioidosis before he left Thailand.

Treatment with ceftazidime (2 g intravenously 4×/d) and trimethoprim/sulfamethoxazole (1,920 mg orally 2×/d) for 4 weeks resulted in gradual defervescence. The patient was discharged with instructions to take trrimethoprim/sulfamethoxazole (1,920 mg orally 2×/d) and doxycycline (100 mg orally 2×/d) for an additional 20 weeks. He returned to Thailand a few weeks after discharge.

*B*. *pseudomallei* infection has been regarded as an occupational hazard for clinical microbiologists (*7**–**9*). Although risk for laboratory-acquired infection is relatively low (*7*), the nature of the disease demands special precautions in dealing with its causative organism. With recent designation of *B*. *pseudomallei* as a select agent by the Centers for Disease Control and Prevention (www.cdc.gov/od/sap), it has been proposed that Biosafety Level 2 practices, which were advised for clinical diagnostic work (*10*), be replaced by stricter safety practices (*11*).

Accordingly, a risk assessment was performed, and 4 persons who had handled the cultures outside a biologic safety cabinet were offered postexposure chemoprophylaxis with trimethoprim/sulfamethoxazole (1,920 mg orally every 8 h) for 3 weeks) (*11*). The frequency of rashes was worrisome: rashes developed in 2 persons (1 elected to complete the course of doxycycline [100 mg orally 2×/d for 3 weeks] and 1 stopped treatment after 10 days). One person was so uncomfortable that she refused further treatment on day 12. Only 1 person completed the course of trimethoprim/sulfamethoxazole without adverse effects. Symptoms consistent with melioidosis did not develop in any of the exposed staff. Serum samples from 10 staff members who had had any contact with the cultures were collected within 2–3 days of exposure and after 6–8 weeks and tested by using an indirect hemagglutination test at Mahidol University in Bangkok. All serologic test results were negative, an outcome consistent with findings of a published report (*8*).

## Conclusions

Many workers (10,600 in 2007) from Thailand have been employed in agriculture in Israel (*12*). Cases of melioidosis may not have been detected until the present patient because routine preemployment medical screenings may have excluded persons with the disease from entry into Israel or unfamiliarity with the disease led to underdiagnosis.

Migration of populations requires awareness regarding unforeseen diseases, as recently highlighted in a clinical problem-solving exercise (*13*). If one considers the abscesses in our patient, melioidosis would have likely been diagnosed earlier had this patient remained in Thailand. In any case, if the routine practice of culturing pus from his abscesses had been followed, the diagnosis might have been made earlier. This finding is a reminder to physicians that they should adhere to basic clinical guidelines. Conversely, clinical evidence and close cooperation of ward physicians, the infectious disease service, and the laboratory staff likely expedited identification of the organism.

The diagnosis of melioidosis in a region where this disease is not endemic depends on physician awareness and laboratory capability. In the patient reported here, clinical suspicion, suggested by multiple visceral abscesses, preceded microbiologic confirmation, which was expedited by internists and the infectious disease service. Clinical microbiology laboratories worldwide should prepare for dealing with *B*. *pseudomallei* and include it in the workup of unusual nonfermentative, colistin-resistant, gram-negative bacilli, particularly in workers from Thailand or other melioidosis-endemic countries. The issue of chemoprophylaxis for persons with laboratory exposures, and its potential for adverse events, requires careful immediate decision making, especially if one considers the rarity of this albeit disabling disease among laboratory staff.
